# Impact of Early Bonding During the Maternal Sensitive Period on Long-Term Effects: A Systematic Review

**DOI:** 10.7759/cureus.53318

**Published:** 2024-01-31

**Authors:** Suresh Babu Mendu, Aruna Rekha Neela, Saritha Tammali, Rakesh Kotha

**Affiliations:** 1 Pediatrics, Government Medical College, Siddipet, Siddipet, IND; 2 Obstetrics and Gynecology, Government Medical College, Siddipet, Siddipet, IND; 3 Pediatrics, Niloufer Hospital, Hyderabad, IND; 4 Neonatology, Niloufer Hospital, Hyderabad, IND

**Keywords:** breast crawl, delivery time, geriatric psychology, neuroendocrine effect, breastfed infant, oxytocin, mother, neonate, bonding, sensitive period

## Abstract

This research project examines the long-term effects of maternal-neonatal bonding during a mother’s “sensitive period.” The review explores how early contact between a mother and her newborn can affect their psychosocial and emotional well-being in the future. Within an hour after birth, oxytocin levels increase for mothers, while catecholamine surges enhance neonates’ memory retention to encourage immediate skin-to-skin contact (SSC), which promotes breastfeeding with benefits, such as quicker placenta expulsion, less bleeding, and lower stress. As per sources to date, there is no systematic review on this subject; however, numerous studies exist regarding short-term outcomes, exclusive breastfeeding, and childhood problems. The exploration involves rigorous searches of academic databases following the Preferred Reporting Items for Systematic Reviews and Meta-Analyses (PRISMA) guidelines for transparency and reproducibility by using the Population, Intervention, Comparison, and Outcome (PICO) framework. Of the 516 initially identified articles, only five were relevant based on refined selection criteria, making it clear from the analysis that sensitive-period bonding produces long-term impacts in infants. Few studies are available, particularly in recent years; thus, more research is required in this area.

## Introduction and background

During the first hour after birth, both the mother and newborn experience a sensitive phase that is programmed by physiological factors, particularly in cases of vaginal delivery [1]. This period benefits from high levels of oxytocin in the mother and exceptionally elevated catecholamines in the infant to facilitate this state [2]. Early contact also plays an impactful role in establishing lasting microbiomes over extended periods [3]. The literature suggests that nurturing maternal bonding during these initial stages significantly influences a child's personal adaptability and versatility [4].

Salk's findings from 1970 indicate that mothers who established early contact with their newborns within the first day of birth displayed a discernible preference for holding them on the left side [5]. Conversely, this inclination was nonexistent among those mothers who interacted with their infants more than one day post-delivery. Mothers who held their babies on the right side had a minimal physical touch and often experienced delayed acknowledgment of parenthood toward their offspring [5].

Animal studies have demonstrated that maternal behavior toward offspring is hormonally mediated. If the mothers do not encounter their young during a critical period, it could result in them rejecting and attacking them for the remainder of their lives [6]. While this was observed in certain animals, like goats and cows, who would reject any separation from their offspring exceeding four hours, such traits were specific to individual species. Conversely, extensive research has been carried out among humans substantiating the existence of sensitive periods [7].

Disorders related to mothering, including child abuse, tend to escalate significantly in cases where there is an early neonatal separation between the mother and the infant, such as in premature births. A study conducted in the United States revealed that mothers who were given the opportunity for early contact with their premature infants displayed distinct variations in their attachment behavior compared to mothers who had their first contact with their infants three weeks after delivery [1].

The purpose of this systematic review is to intentionally examine the correlation between initial maternal and infant attachment and the long-lasting effects on infants.

## Review

Objective

The primary focus of this review is to assess the effects on mother-child interaction when close contact between them is established during a critical period after birth, commonly known as the sensitive period. 

Methods

**Figure 1:**
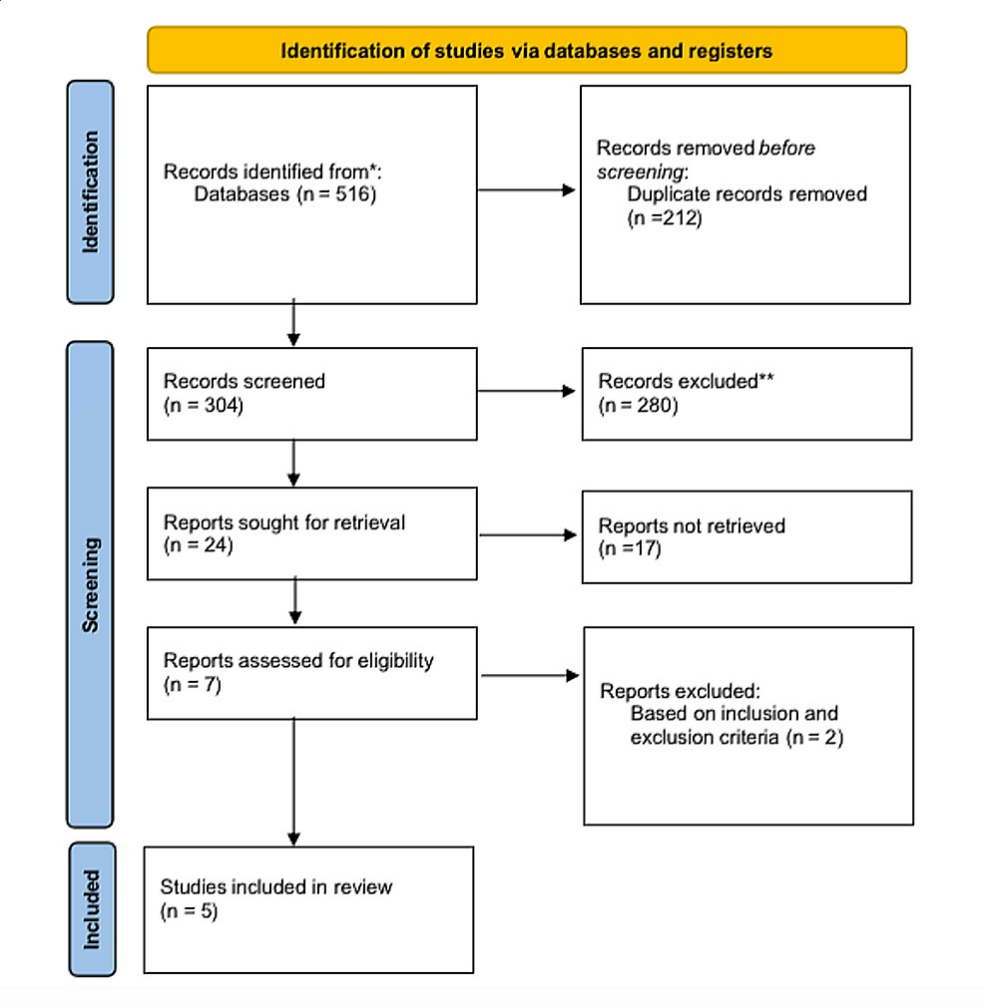
Preferred Reporting Items for Systematic Reviews and Meta-Analyses (PRISMA) diagram

The study’s search is dependent on specific methods to be examined. The approach involves conducting systematic searches using major academic databases, such as PubMed, Embase, and Scopus, for relevant research on the relationship between early maternal bonding and psychosocial-emotional problems in infants. To generate useful search terms, a combination of keywords encompassing “early maternal bonding,” “child psychosocial development,” “mother-sensitive period,” “breast crawl,” and “mother and child bonding” was used. We derived medical subject headings (MeSH) using thematic keywords. Utilizing boolean operators (AND/OR) can refine or broaden these keyword combinations when necessary by eliminating studies outside certain age groups or unsuitable outcomes. We used asterisks *, parentheses (), and field codes. We searched both the text and the abstracts. To ensure scientific rigor throughout the exploration process with regard to transparency, replicability, and meticulousness of execution, we followed guidelines from Preferred Reporting Items for Systematic Reviews and Meta-Analyses (PRISMA) (Figure 1). Furthermore, utilizing the Population, Intervention, Comparison, and Outcome (PICO) framework is an essential guide for literature searches (Table 1). The International Prospective Register of Systematic Reviews (PROSPERO) has duly registered our methodology under ID 501742.

**Table 1:** Population, Intervention, Comparison, and Outcome (PICO) framework

PICO framework	
Population	Neonate and children
Intervention	Early maternal bonding during the sensitive period
Comparison	Lack of early maternal bonding experiences
Outcome	Presence and severity of long-term bonding between the mother and child
Study	Observational and randomized studies

Study Identification

A comprehensive search was conducted utilizing the EndNote device on major academic databases, namely, PubMed, Embase, and Scopus. The combination of keywords mentioned previously was used to identify relevant research exploring the correlation between early maternal bonding and future mother-child interaction. Subsequent to this initial search, a meticulous manual review was carried out on the articles to ensure that no duplicates were present. This measure ensured that each article would only be considered once in subsequent phases of the systematic screening process, with predetermined screening criteria aimed at filtering studies focused specifically on the relationship between neonatal bonding and attachment issue development. Finally, we also examined all references cited in the selected articles for inclusion.

Data Extraction

The chosen research gathered pertinent information regarding our study topic, with a focus on the study’s design, location, sample size, and outcomes. To ensure that only suitable articles were included in the specific review process, the available research adhered to inclusion criteria.

Inclusion and Exclusion Criteria

Only peer-reviewed research published in English and included in PubMed, Scopus, and Embase was considered for the systematic review. We included observational studies and randomized trials focusing on maternal-fetal interactions after contact in the sensitive period. Due to the limited number of studies available, we did not set a time limit for our search criteria. Articles that included under-18-year-old children were included. Editorial commentary articles, opinion articles, conference papers, abstracts lacking comprehensive data and results, and incomplete articles were excluded. Articles that focused primarily on early outcomes and did not meet the objectives of our review, including articles that included children older than 18 years, were excluded.

Results

In the initial stage of our research project, we conducted a comprehensive search and identified 516 articles that were pertinent to exploring the impact of early maternal bonding on child psychosocial disorders. After completing an extensive review, 212 sources were recognized as duplicates and subsequently removed from consideration, resulting in 304 articles for further assessment. As the screening progressed, we found that an additional set of 280 articles did not align with our exploration objectives; hence, we eliminated them. Eventually, 24 relevant publications met our criteria, of which seven came forward for evaluation. After scrutinizing those articles against the inclusion-exclusion framework specifications outlined earlier, two were disqualified, leaving us with five credible selective papers [5].

Assessment of Quality

The Newcastle-Ottawa scale evaluates the quality of research studies based on three main criteria: group selection, comparability of the groups, and outcome assessment. The scale consists of four sections, namely, representation of the study, selection of the study participants, ascertainment of exposure, and demonstration of the outcome of interest. Each of these sections is assigned a maximum of one star, indicating the level of quality.

The comparability section focuses on three specific questions aimed at determining the extent to which the study controls for socioeconomic status (SES), any additional factors, or an insufficient level of control. Each question is given a maximum of one star, reflecting the degree of comparability achieved.

The outcome assessment section involves evaluating the quality of the outcome by examining whether it is supported by reliable records, independent studies, or direct observations. It also considers factors, such as record linkages, cases of self-reporting (particularly when original medical records are not referenced), and the presence or absence of outcome descriptions. Each aspect of this assessment is awarded a maximum of one star, indicating the quality of the outcome assessment.

Version 2 of the Cochrane risk-of-bias tool for randomized trials (RoB 2) offers a comprehensive approach to evaluating the potential bias in the results of randomized trials across various types of research. It presents a systematic assessment that encompasses several domains where bias could potentially arise within a trial. It is important to note that all five domains are obligatory components of the assessment process.

Due to the unique study designs and research objectives of each article, it is important to use quality evaluation techniques judiciously. For observational studies, we utilized the Newcastle-Ottawa scale and found all articles to be of good quality. Meanwhile, for randomized controlled studies, we employed RoB 2. By employing these assessment tools, we were able to confirm the validity of our sources based on their chosen research type. According to the RoB 2 analysis results, two randomized control trials had a low risk-bias rating (Table 2).

**Table 2:** Quality assessment of observational studies

SN	Study	Selection	Comparability	Outcomes
1	de Chateau et al. (1977) [7]	***	**	***
2	Widström et al. (2019) [8]	***	**	***
3	Hales et al. (2008) [9]	***	**	***

Summary of the Selected Studies 

In accordance with the PRISMA methodology, five studies were included in this review (Table 3). Lactation is a natural cycle present in all mammalian species that results from developmental forces creating an optimal nutrient delivery system, facilitating the provision of essential nutrients to offspring by mothers in adequate amounts. The authors highlighted the examination of current findings regarding the psychological impacts of breastfeeding on both infants and their mothers [7].

**Table 3:** Summary of the selected articles

S. No.	Authors	Study type	Country	Condition/outcome
1	de Chateau et al. (1977) [7]	Observational study	Sweden	More smiles and fewer cries in the early skin-to-skin contact group
2	Bystrova et al. (2009) [10]	Randomized controlled trial	Germany	Maternal sensitivity, infant self-regulation
3	Widström et al. (2019) [8]	Observational study	Sweden	Behavior of the healthy, alert, full-term infant placed skin to skin
4	Hales et al. (2008) [9]	Observational study	USA	The objective of this research was to study the influence of birth routines on mother-infant interaction.
6	Dumas et al. (2013) [11]	Randomized controlled trial	USA	Results also show evidence of a sensitive period for separation after birth.

*Description of Studies* 

According to de Chateau's case-control study, mothers giving birth for the first time and infants receiving an additional 15-20 minutes of SSC within the initial hour after birth showed a stronger bond at 36 hours old [7]. The study followed these individuals through direct observation during free play sessions and interviews with the mothers over three months. The results revealed that those in this extra contact group spent more time kissing their babies while making eye contact, leading to more smiles from them; babies also cried less frequently. Interestingly, this effect was even stronger among mother-boy pairs than mother-girl ones [7].

According to Ksenia Bystrova, MD, having SSC and initiating breastfeeding within the first two hours after birth can have a positive impact on parent-child early relationship assessment [10]. This includes increased maternal sensitivity, improved infant self-regulation, and enhanced dyadic reciprocity between parent and child. The study also found no compensating factor for the negative effects of separating mother and child for two hours following childbirth, even if rooming-in is practiced afterward [10]. According to Widström and colleagues [8], newborn infants have a sensitive period that aligns with the mother's sensitivity within the first hour of birth. In their research, they identified nine stages of breast crawling by analyzing videos. This study contributed to implementing uninterrupted SSC immediately after delivery based on clinical observations. The findings indicated that such contact during this critical time positively impacted infant self-regulation and improved mutual relationships between mothers and infants one year later [8].

A study conducted by Hales et al. [9] at Roosevelt Hospital in Guatemala City examined the effects of early postpartum contact between mothers and their newborns. The participants were 60 healthy first-time mothers aged 16 to 35 years who had undergone routine vaginal deliveries. Their research focused on observing behaviors, such as proximity maintenance, affectionate behavior, and caretaking tendencies among both groups: those with early contact versus delayed contact with their infants after childbirth. The results showed that these behaviors were significantly higher among those within the former group. Notably, there was a significant statistical difference (p < 0.01) found in affectionate behavior [9]. 

According to a study by Dumas et al. [11], mothers displayed harsh behaviors toward their infants on the fourth day after birth if they were separated from them and placed in a crib immediately. The findings indicated that there is a crucial period for separation following birth, as demonstrated by comparing SSC and nursery groups during this timeframe. Two variables showed significant differences between the two groups: attempts at breastfeeding (p = 0.010, 2df) and wakefulness most of the time period (p = 0.010, 3df). Mothers who had constant SSC with their newborns exhibited more effort in trying to have them latch onto breast milk while in cribs; however, those whose babies were put into cribs struggled more frequently with nursing issues. The newborns lacked attention, and their maternal figures displayed a significant amount of harsh behavior toward them [11].

Discussion

Establishing profound bonds for the future is imperative during the early stages of neonatal-maternal interaction. This study delves into the intricate dynamics of initial mother-child bonding and its long-term effects on a child’s psychosocial and emotional development [12]. Following birth, there is an increase in maternal serum gastrin, oxytocin, and prolactin levels, indicating extensive neuroendocrine communication between both parties, which plays a crucial role among animal offspring [13]. The breast crawl phenomenon witnessed in human babies results from SSC with their mothers’ chests as they instinctively initiate breastfeeding independently like other animals [14-17]. Unfortunately, many healthcare providers within maternity services lack exposure to guidelines about initiating breastfeeding 30 minutes post-birth, reflecting unverified efficiencies that interfere drastically while adhering to these recommendations [17]. Research studies reported by Nandi et al. identified small academic gains associated exclusively with boys through extended periods of breastfeeding interventions compared to girls residing in India [18]. In his primary research, Condon and Sander [19] asserted that newborn humans move in coordinated and consistent manners when crawling, mirroring the structure of adult speech. This discovery suggests that infants engage interactively with their surroundings from an early stage rather than being solitary beings [19]. Another study by Righard et al. involved 72 babies born naturally: one group was separated from their mothers, while another practiced breast crawling accompanied by physical contact [20]. The researchers monitored them for two hours after birth and found more correct sucking techniques among those in the latter (24 out of 38) compared to those experiencing separation (seven out of 34) [20].

Marshall conducted a case-control study on 28 primiparous mothers, with 14 participants in each group [21]. Maternal behavior was observed between the ages of 28 and 32 days postpartum. Results showed that mothers with early contact exhibited more eye contact, fondling, and comforting behaviors toward their infants than those without early contact [21]. In her pilot study, Schroeder investigated two groups of 10 primiparas [22]. One group had access to rooming-in while the other resided in a maternity ward setting. Data were collected through an open-ended questionnaire. The findings demonstrated that mothers who practiced rooming-in spent more time bonding with their babies, resulting in stronger connections through thoughts and emotions toward their newborns, as measured by the study’s outcome [22].

In a randomized controlled trial with parallel groups, Cooijmans et al. [23] conducted an experiment involving 116 mothers and their infants born at full term. The intervention began immediately after birth, lasting for five weeks. The participants in the control group were not required to engage in SSC. Maternal and infant outcomes were evaluated at intervals of two, five, and 12 weeks postpartum and one year after delivery. Results indicated that SSC had a positive effect on reducing maternal postpartum depressive symptoms while also yielding numerous benefits, such as a quicker growth rate among infants, leading to better health conditions. They tended to cry less often and sleep more soundly. Mothers likewise experienced lower levels of anxiety or stress resulting from greater involvement during breastfeeding periods, which led them to cultivate stronger bonds with their newborns [23]. Moore et al. systematically reviewed 34 randomized trials involving 2,177 mother-baby pairs [24]. The review demonstrated early bonding’s significant positive impact on breastfeeding at one to four months postpartum, with a risk ratio of 1.27 and a confidence interval between 1.06 and -1.53. In addition, SSC increased the duration of breastfeeding by an average mean difference of 42.55 days, although this did not quite reach statistical significance (p = 0.06). Early bonding also resulted in enhanced cardiorespiratory stability and high blood glucose levels for late preterm infants compared to cases where no such interaction occurred [24]. Unlike the above studies, Widström et al. found that there were no differences between intervention groups 10 months after birth; however, they noted positively influencing mother-infant interactions through the infant’s initial touch with their areola or nipple within the first four days following delivery [25]. In their review in 1982, Hobert et al. did not support the concept of a sensitive period and its long-term effects [26]. The acceptability of breast crawls, particularly in developing countries, is a significant concern because of the busy labor rooms and a lack of manpower [27].

## Conclusions

Breast crawling during the mother’s sensitive period has been proven to provide physical and emotional benefits for mother and child, but unfortunately, it is not widely practiced in many countries. It is crucial that intact survival is important, which was a concept examined in studies long ago. Although sensitive periods have been previously researched, a limited amount of modern investigation has been conducted in this field. However, most past research supports the notion that introducing a sensitive period would improve mother-infant outcomes over time. Numerous studies centering around breastfeeding and neonatal futures also exist.

Our review focused on how implementing this sensitivity window could enhance bonding between infants and caregivers while reducing the risks of psychosocial or emotional instabilities later in life. In light of these findings, we strongly advise promoting immediate post-delivery bonding experiences without delay across all medical facilities whenever possible. It is highly recommended that investigations be conducted on the effects of sensitive periods in adults.
